# Reference standards for lean mass measures using GE dual energy x-ray absorptiometry in Caucasian adults

**DOI:** 10.1371/journal.pone.0176161

**Published:** 2017-04-20

**Authors:** Mary T. Imboden, Ann M. Swartz, Holmes W. Finch, Matthew P. Harber, Leonard A. Kaminsky

**Affiliations:** 1 Ball State University, Muncie, Indiana, United States of America; 2 University of Wisconsin-Milwaukee, Milwaukee, Wisconsin, United States of America; TNO, NETHERLANDS

## Abstract

Body composition assessments commonly focus predominantly on fat mass, however lean mass (LM) measurements also provide useful information regarding clinical and nutritional status. LM measurements help predict health outcomes and diagnose sarcopenia, which has been associated with frailty. Dual energy x-ray absorptiometry (DXA) is an established technique used in clinical and research settings to assess body composition including total and regional LM. Currently, there are no reference values available that were derived from GE-Healthcare DXA systems directly for US adults for LM, LM index (LMI), percent LM (%LM), and appendicular lean mass index (ALMI) and it is known that whole-body and regional LM measures differ by DXA manufacturer.

**Objective:**

To develop reference values by age and sex for LM measures using GE-Healthcare DXA systems.

**Methods:**

A de-identified sample was obtained from Ball State University’s Clinical Exercise Physiology Laboratory and University of Wisconsin-Milwaukee’s Physical Activity & Health Research Laboratory. DXA scans of 2,076 women and 1,251 men were completed using a GE Lunar Prodigy or iDXA. Percentiles (%ile) were calculated for all variables of interest (LM, LMI, %LM, and ALMI) and a factorial ANOVA was used to assess differences for each variable between 10-year age groups and sex, as well as the interaction between age and sex.

**Results:**

Men had higher mean total LM, %LM, LMI, and ALMI than women (p<0.01), across all age groups. All LM variables decreased significantly over the 5 decades in men, however in women only total LM, %LM, and ALMI decreased from the youngest to oldest age groups (p<0.01).

**Conclusion:**

These reference values provide for a more accurate interpretation of GE-Healthcare DXA-derived LM measurements offering clinicians and researchers with an initial resource to aid in the early detection and assessment of LM deficits.

## Introduction

Body composition assessments commonly focus on fat mass measurements, however lean mass (LM) also provides useful information regarding an individual’s health and nutritional status. LM plays an important role in helping to maintain bone density, improve metabolic health, preserve strength, and reduces the risk of injury and falls [1]. Additionally, significant reductions in LM with age or accompanying disease, known as sarcopenia and cachexia, have been associated with decreased function and quality of life, as well as the development of frailty [1, 2]. Therefore, body composition assessments across the life span that measure LM are valued in clinical and research settings, to aid in the identification and diagnosis of sarcopenia and cachexia. Computed tomography (CT) and magnetic resonance imaging (MRI) are considered gold standards for measuring whole body LM, as they have shown excellent measurement accuracy when compared to cadaver analysis (r = 0.99) [3]. However, they are impractical in most clinical and research settings due to their high cost and radiation exposure. LM measured by dual energy x-ray absorptiometry (DXA) is a composite of non-fat and non-bone tissue and is highly associated with LM measured using CT and MRI technology (r^2^ = 0.86–0.96) [4–6]. DXA is also less expensive and time-consuming than CT and MRI scans, and therefore is considered the best alternative method for measuring LM [4, 7].

DXA is a three-compartment method that distinguishes total bone mineral content from soft tissue with high precision and accuracy, dividing the latter into fat and lean body mass. DXA is also advantageous over other body composition methods due to its ability to assess both total and regional body composition. This allows the assessment of whole-body and site specific LM. From these measurements total LM, LM index (LMI), percent LM (%LM), and appendicular LM index (ALMI) can be determined, all of which have been used previously in research and clinical settings to diagnose sarcopenia or identify those at risk of physical disability [1, 7–11]. However, since there are no universally recognized standards for these key LM variables using GE-Healthcare DXA systems in healthy US adults (Total LM, LMI, %LM, and ALMI) it makes the interpretation of results challenging.

In 2009, Kelly et al. used data from the National Health and Nutrition Examination Survey (NHANES) to develop reference values for measures of LM specific to DXA measurement obtained with the Hologic QDR 4500A fan beam densitometer [12]. Hologic and GE-Healthcare are the two dominant DXA manufacturers, which have been validated against criterion 4-compartment models [13, 14]. However these devices have been shown to produce different body composition results, including differences in whole-body and regional LM measurements [15]. These discrepancies in measurements between manufacturers may be due to differences in instrumentation, fan beam angle, calibration standards, and manufacturer derived algorithms used to calculate the body composition measurements. Shepherd et al. compared the body composition results from the Hologic and GE-Healthcare Lunar DXA systems and found significant differences between the Hologic and GE-Healthcare devices for total LM and ALMI (23.2 vs. 21.8 kg and 9.5 vs. 8.7 kg, respectively p<0.01) measured in adults [15]. Similar differences in LM measurements between DXA manufacturers have been reported in the literature [16, 17].

Fan et al. used cross-calibrated equations between the Hologic and GE-Healthcare models to convert whole-body and regional bone and soft tissue measurements from the NHANES 1999–2004 dataset to reference values for the GE-Healthcare models [18]. Although this study provided an initial set of reference values for body composition measures, including total LM, LMI, and ALMI for GE-Healthcare DXA models, these are estimates and therefore it is important to develop body composition reference values obtained directly from whole-body scans using the GE-Healthcare models [15]. These standards are needed to appropriately determine values for body composition measurements that are associated with LM deficits that can predict poor health-outcomes and lead to decreased physical function.

Other DXA-derived measures of LM are useful in predicting health-risks associated with body composition. LMI is a measure of total skeletal muscle LM (kg) scaled to height (m^2^). This index allows for the comparison of LM body composition in individuals of different height, as it is not confounded by fat mass, making it a useful tool for assessing LM deficits. Additionally, ALMI, calculated as the sum of arm LM and leg LM scaled to height (m^2^), is thought to be a surrogate of skeletal muscle mass and therefore is another important measure used to identify and assess the development of sarcopenia [1]. Reference values for LMI and ALMI have been developed in Australian and Mexican adult populations using direct measurement from GE-Healthcare DXA models, however these LM reference values may not be consistent for US adults as a result of differences in geographical regions [7, 19].

As GE-Healthcare is one of the two major DXA manufacturers [15] widely used by researchers and clinicians, reference values are needed to guide interpretation of body composition results obtained with this instrument. The purpose of this study was to develop reference values for LM variables including total LM, LMI, %LM and ALMI for a US population using the GE-Healthcare DXA models.

## Methods

A de-identified sample of 3,327 participants (2,076 women, 1,251 men; 95% of which were Caucasian), was obtained from Ball State University’s Clinical Exercise Physiology Laboratory (2,218 scans) and University of Wisconsin-Milwaukee’s Physical Activity & Health Research Laboratory (1,109 scans). Participants were either self-referred, residents of the surrounding communities, or research subjects or participants at one of the two laboratories, all of which were considered apparently healthy. All participants were ≥20 and <80 years old (mean age 45.8 ± 18.3 years (**Table 1**)). Only the first scan was used in analysis for individuals with repeat scans. Participants were excluded if their width exceeded the scanner field or their body weight exceeded the limits of the scanner bed (159 kg (350 lbs) for Prodigy or 204 kg (450 lbs) for iDXA). The study was declared exempt by the Ball State University and University of Wisconsin-Milwaukee Institutional Review Boards as all data was de-identified. Additionally, prior to all DXA scans participants signed an informed consent agreeing that their data from the scan could be used for research purposes.

**Table 1:** Descriptive characteristics of participants by gender (Mean ± SD).

	**Men (n = 1,251)**	**Women (n = 2,076)**
**Age (yr)**	46.1 ± 19.1	45.6 ± 17.9
**Ethnicity**	92.2% Caucasian	96.1% Caucasian
**Height (cm)**	177.8 ± 7.6	164.8 ± 6.9*
**Weight (kg)**	89.1 ± 18.6	70.9 ± 18.4*
**BMI (kg m^**-2**^)**	28.0 ± 5.3 Distribution • Underweight 0.5% • Normal 31.5% • Overweight 41.2% • Obese 26.8%	26.4 ± 6.7* Distribution • Underweight 0.7% • Normal 47.0% • Overweight 27.0% • Obese 25.3%
**Total body fat (%)**	28.3 ± 8.3	38.6 ± 8.9*

* Significantly different from Men (p<0.01).

BMI distribution based on the World Health Organization classification.

A whole-body DXA scan was performed on all participants. The GE-Healthcare Lunar Prodigy was used at the Clinical Exercise Physiology Laboratory at Ball State University from 2003 to 2010 and the GE-Healthcare iDXA was used from 2010 to October 2015. All scans performed at the Physical Activity & Health Research Laboratory at the University of Wisconsin-Milwaukee used the GE Lunar Prodigy from 2005 to October 2015. Both the GE-Healthcare Lunar Prodigy and iDXA are narrow fan-beam densitometers that use the enCORE software platform that has been updated as GE-Healthcare releases new versions (Ball State University: 13.40.038, University of Wisconsin-Milwaukee: 13.31.016). These two GE-Healthcare DXA models have been shown to have high agreement between systems (R^2^ = 0.85–0.99) for both total and regional lean mass measurements, thus are suitable for intra-subject comparisons [20, 21, 22].

### Procedure

A full explanation of the procedures have been described previously [23]. In summary DXA scans were administered by trained research technicians using standardized procedures recommended by GE-Healthcare. All technicians were trained over a period of 1 to 3 months at each laboratory. Prior to each testing session, the GE-Healthcare DXA systems at both laboratories were operated following manufacturer guidelines, passing the recommended quality assurance procedure. Participants were asked to remove all metal, as well as shoes prior to the scan and their height was measured using a wall-mounted stadiometer and mass using a calibrated scale. The participant was then positioned according to manufacturer specifications within the scanner field on the DXA table. Automatic scan mode and automatic analysis mode were used as the default setting at both sites and one trained technician at each site analyzed the DXA scans. Technicians modified the ROI only if determined to be significantly off. Variables of interest from the scan that were used in this analysis included LM, leg LM, and arm LM [24]. Leg LM and arm LM were summed to calcuate appendicular lean mass.

### Statistical analysis

All statistical analyses were performed using SPSS (version 24.0), with descriptive measures reported as means ± standard deviation. Sex- and age-specific body composition measurements were analyzed, with participants classified into age groups by decade (20–29, 30–39, 40–49, 50–59, 60–69, and 70–79 years). The distribution of men and women between decades was checked for normality using the Kolmogorov-Smirnov test. This distribution was found to be non-normal (p<0.05). The LM, LMI (LM (kg) height (m^-2^)), %LM, ALMI ((arm LM + leg LM) (kg) height (m^-2^)) [25] were calculated from scan measurements. Percentiles were calculated for each outcome variable specific to sex and age groups. A factorial ANOVA was used to determine potential differences in mean LM, LMI, %LM, ALMI, between sex- and age-specific groups. Finally, reference curves were created using LMS regression (S1 Fig–S8 Fig), where for each variable of interest, the model was fit using a B-spline with a difference penalty. The curves were fit to the 3^rd^, 50^th^, and 97^th^ percentiles superimposed upon the raw data values [26]. The median values from the NHANES cohort were also added to these curves for comparison. An alpha level was set at 0.05 to determine statistical significance.

## Results

The demographic information from the sample is shown in **Table 1**. The mean age of the sample was 45.8 ± 18.3 years. Mean BMI, weight, height, and percent body fat were significantly different between sexes (p<0.05). Mean BMI, Weight, and percent body fat significantly increased from the youngest (20–29 years) to oldest (70–79 years) age groups in women (23.8 to 27.2 kg m^-2^; 66.2 to 70.3 kg; 31.4 to 40.4%, respectively; p<0.05). In men, BMI significantly increased between the youngest (20–29 years) and the 2^nd^ to oldest age group (60–69 years) (26.5 to 28.8 kg m^-2^, p<0.05). Whereas weight significantly decreased, but percent body fat significantly increased from 20–29 to the 70–79 year age group in men (87.1 to 83.4 kg; 21.1 to 31.1%, respectively; p<0.05).

Mean (± SD) and percentiles of LM from the GE-Healthcare models by age for both women and men are displayed in **Table 2**. Men had a greater mean LM than women (men: 60.8 kg, women: 42.3 kg; p<0.01), across all age groups. The mean LM decreased significantly with increasing age in both men and women from the youngest to oldest age groups (p<0.05). In men it was observed that mean LM decreased with increasing age up until 70–79 years, however in women mean LM remained constant until the 5^th^ decade, after which it starts to decline (p<0.05).

**Table 2:** Sex-specific percentiles of total lean body mass (kg) measured with DXA.

	**Women**										
**AGE (YR)**	n	X ± SD	10^th^	20^th^	30^th^	40^th^	50^th^	60^th^	70^th^	80^th^	90^th^
**20–29**	562	43.0± 6.1	35.7	37.6	39.3	41.0	42.5	43.7	45.5	47.7	51.1
**30–39**	196	43.6± 6.8	36.3	38.3	39.6	41.3	42.5	43.8	45.9	48.7	52.4
**40–49**	258	42.9±7.0	35.4	36.9	38.4	39.9	41.6	43.0	44.8	47.5	51.8
**50–59**	437	42.3±6.9 ^C^	34.4	36.4	38.1	39.7	41.5	43.1	45.1	47.2	51.5
**60–69**	440	40.8± 5.5 ^B^	34.9	36.5	37.9	39.1	40.8	42.1	43.6	45.4	48.2
**70–79**	183	39.0± 5.0 ^A^	32.9	34.9	36.2	37.9	39.1	40.4	41.6	43.1	45.1
	**Men**										
**AGE** **(YR)**	n	X ± SD	10^th^	20^th^	30^th^	40^th^	50^th^	60^th^	70^th^	80^th^	90^th^
**20–29**	384	65.0±11.0 ^D^	53.5	57.0	59.1	61.4	63.9	66.2	69.5	73.6	82.0
**30–39**	104	60.9±9.0 ^E^	52.1	55.1	56.5	59.8	61.8	63.7	67.3	69.8	74.1
**40–49**	145	60.2±8.5 ^E^	51.4	53.9	56.8	58.4	60.2	62.1	64.0	67.9	73.2
**50–59**	214	59.3±8.1	50.5	53.0	55.3	57.5	60.5	61.8	63.8	65.8	70.2
**60–69**	236	58.9±6.9	50.2	53.2	55.7	57.5	58.5	60.2	61.9	63.9	67.9
**70–79**	168	54.3±6.0	47.6	49.8	51.4	52.9	54.2	56.0	57.4	59.8	62.3

^A^ Significantly different than 20–29, 30–39, 40–49, 50–59, 60–69; p<0.05.

^B^ Significantly different than 20–29, 30–39, 40–49, 70–79; p<0.05.

^C^ Significantly different than 30–39, 60–69, 70–79; p<0.05.

^D^ Significantly different than 30–39, 40–49, 50–59, 60–69, 70–79; p<0.05.

^E^ Significantly different than 20–29, 50–59, 60–69, 70–79; p<0.05.

LMI means (±SD) and percentiles by age group for both women and men are presented in **Table 3**. Mean LMI was higher in men than women at all age groups (men: 19.2 ±2.4 kg m^-2^; women: 15.7± 2.2 kg m^-2^, p < 0.05). Additionally, it was observed that the mean LMI decreased across each decade in men, whereas in women mean LMI appeared to increase up until 40–49 years of age after which a decrease was observed.

**Table 3:** Sex-specific percentiles of lean mass index (kg m^-2^) measured with DXA.

	**Women**										
**AGE (YR)**	n	X ± SD	10^th^	20^th^	30^th^	40^th^	50^th^	60^th^	70^th^	80^th^	90^th^
**20–29**	562	15.5± 1.8 ^A^	13.4	14.0	14.5	14.9	15.3	15.6	16.2	17.0	18.0
**30–39**	196	15.8± 2.2	13.3	14.1	14.6	15.0	15.4	16.1	16.7	17.6	18.8
**40–49**	258	15.9±2.5 ^B^	13.2	13.8	14.4	14.9	15.3	15.9	16.5	17.3	19.0
**50–59**	437	15.8±2.2	13.2	13.9	14.4	15.0	15.5	16.0	16.6	17.3	18.7
**60–69**	440	15.4±2.1	13.3	13.9	14.4	14.8	15.3	15.7	16.4	17.0	18.2
**70–79**	183	14.9±1.8 ^C^	13.2	13.8	14.2	14.4	14.9	15.3	15.8	16.7	17.7
	**Men**										
**AGE** **(YR)**	n	X ± SD	10^th^	20^th^	30^th^	40^th^	50^th^	60^th^	70^th^	80^th^	90^th^
**20–29**	384	19.8± 2.7 ^D^	16.9	17.8	18.3	19.0	19.5	20.3	21.4	22.3	23.8
**30–39**	104	19.0±2.2	16.8	17.4	18.2	19.0	19.3	19.7	20.2	21.2	22.2
**40–49**	145	18.9±2.2	16.7	17.4	17.9	18.6	19.2	19.6	20.3	21.0	22.1
**50–59**	214	18.7±2.1	16.3	17.2	17.8	18.3	18.8	19.4	20.1	20.8	21.6
**60–69**	236	18.9±2.0	16.8	17.3	17.8	18.3	18.9	19.3	19.8	20.4	21.3
**70–79**	168	17.8±1.7^C^	16.2	16.8	17.1	17.4	17.7	18.0	18.4	19.0	19.9

^A^ Significantly different than 30–39, 40–49, 50–59; p<0.05.

^B^ Significantly different than 20–29, 60–69, 70–79; p<0.05.

^C^ Significantly different than 20–29, 30–39, 40–49, 50–59, 60–69; p<0.05.

^D^ Significantly different than 40–49, 50–59, 60–69, 70–79; p<0.05.

Mean (± SD) and percentiles of %LM from the GE-Healthcare models by age for both women and men are displayed in **Table 4**. Men had a greater mean %LM than women (69.6 ± 8.8% vs. 59.3 ± 9.3%, respectively; p<0.01), across all age groups. The mean %LM decreased with increasing age in men until the 60–69 year age group (p<0.05), where it then remained steady. In women, mean %LM decreased up until the 50–59 year age group (p<0.05) and then remained steady in the 60–69 year age group, where thereafter mean %LM significantly increased from the 60–69 to70-79 year age group (p<0.05).

**Table 4:** Sex-specific percentiles of % lean body mass measured with DXA.

	**Women**										
**AGE (YR)**	n	X ± SD	10^th^	20^th^	30^th^	40^th^	50^th^	60^th^	70^th^	80^th^	90^th^
**20–29**	562	65.7± 8.1 ^A^	54.4	59.4	62.3	65.4	67.4	69.3	71.1	72.9	76.1
**30–39**	196	61.0±10.3 ^B^	47.7	51.6	54.2	57.2	60.6	63.6	67.9	70.9	75.1
**40–49**	258	58.6±9.0 ^C^	46.4	49.3	52.8	54.8	57.4	59.8	62.8	65.6	71.5
**50–59**	437	56.3±8.1	46.5	49.2	51.4	53.3	55.3	57.5	59.7	63.1	66.8
**60–69**	440	56.2±7.2	46.5	49.2	51.4	53.3	55.6	57.1	59.2	62.1	64.9
**70–79**	183	58.6±7.5 ^C^	48.1	50.3	53.0	55.1	57.1	58.9	60.6	63.8	67.6
	**Men**										
**AGE** **(YR)**	n	X ± SD	10^th^	20^th^	30^th^	40^th^	50^th^	60^th^	70^th^	80^th^	90^th^
**20–29**	384	75.7±7.8 ^A^	65.3	68.7	71.8	74.3	77.0	78.6	80.4	83.1	86.4
**30–39**	104	72.6±9.9 ^B^	58.2	62.2	64.3	67.4	71.0	73.8	77.2	80.2	85.2
**40–49**	145	69.1±8.1	57.5	61.0	64.1	66.0	67.5	69.1	71.7	74.6	80.2
**50–59**	214	67.2±7.3	57.6	59.8	62.0	63.8	65.6	67.5	70.1	72.4	76.6
**60–69**	236	66.8±7.3	57.1	60.6	63.0	64.3	64.9	67.6	69.7	72.0	75.5
**70–79**	168	67.0±6.2	58.2	60.5	62.5	64.4	66.1	68.3	69.3	72.1	74.3

^A^ Significantly different than 30–39, 40–49, 50–59, 60–69, 70–79; p<0.05.

^B^ Significantly different than 20–29, 40–49, 50–59, 60–69, 70–79; p<0.01.

^C^ Significantly different than 20–29, 30–39, 50–59, 60–69; p<0.01.

ALMI means (±SD) and percentiles by age group for both women and men are presented in **Table 5**. Mean ALMI was higher in men than women (8.8± 1.4 kg m^-2^ vs. 6.8 ±1.1 kg m^-2^; p< 0.05) across all age groups. Additionally, in both sexes ALMI decreased over the 5 decades, as the mean for men and women aged 20–29 years decreased from 9.47 and 6.96 to 7.94 and 6.33 for age 70–79 years, respectively (p<0.05). The decrease in ALMI can be attributed to a reduction in arm LM of 4.1% and 2.6% and reductions in leg LM of 5.2% and 2.8% in men and women, respectively.

**Table 5:** Sex-specific percentiles of appendicular lean mass index (kg m^-2^) measured with DXA.

	**Women**										
**AGE** **(YR)**	n	X ± SD	10^th^	20^th^	30^th^	40^th^	50^th^	60^th^	70^th^	80^th^	90^th^
**20–29**	562	6.96±0.97	5.8	6.1	6.4	6.7	6.9	7.1	7.4	7.8	8.3
**30–39**	196	6.95±1.10	5.7	6.1	6.4	6.6	6.8	7.1	7.4	7.8	8.4
**40–49**	258	6.88±1.20	5.7	5.9	6.1	6.3	6.7	7.0	7.2	7.6	8.2
**50–59**	437	6.71±1.10 ^A^	5.5	5.8	6.1	6.3	6.6	6.8	7.1	7.4	8.0
**60–69**	440	6.62±1.00^A^	5.6	5.9	6.1	6.3	6.5	6.8	7.1	7.4	8.1
**70–79**	183	6.33±0.87 ^B^	5.3	5.7	5.9	6.1	6.3	6.5	6.7	7.1	7.5
	**Men**										
**AGE** **(YR)**	n	X ± SD	10^th^	20^th^	30^th^	40^th^	50^th^	60^th^	70^th^	80^th^	90^th^
**20–29**	384	9.47±1.56 ^C^	7.9	8.3	8.6	9.0	9.3	9.6	10.2	10.9	12.1
**30–39**	104	8.94±1.20	7.7	8.1	8.6	8.9	9.1	9.3	9.6	10.0	10.6
**40–49**	145	8.75±1.17	7.3	8.0	8.3	8.5	8.7	9.0	9.2	9.8	10.5
**50–59**	214	8.53±1.11 ^A^	7.4	7.8	8.1	8.4	8.6	8.9	9.2	9.5	10.1
**60–69**	236	8.48±1.01 ^A^	7.4	7.7	8.0	8.3	8.5	8.8	9.0	9.3	9.7
**70–79**	168	7.94±0.86 ^B^	7.1	7.4	7.6	7.8	8.0	8.2	8.3	8.6	9.1

^A^ Significantly different than 20–29, 30–39, 40–49, 70–79; p<0.05.

^B^ Significantly different than 20–29, 30–39, 40–49, 50–59, 60–69; p<0.05.

^C^ Significantly different than 30–39, 40–49, 50–59, 60–69, 70–79; p<0.05.

## Discussion

The current research represents the first reference values for total LM, LMI, %LM, and ALMI using measures obtained directly from GE-Healthcare DXA systems for adults in the United States. The only other known reference values for these LM variables measured directly from DXA were derived from the 1999–2004 NHANES dataset [12], which used the Hologic QDR 4500A system that is known to differ from GE-Healthcare systems [15], as well as in reference datasets from Mexican [19] and Australian [7] adult populations.

Total LM is an important indicator of muscle mass and has been found to be associated with muscular strength and dependency in activities, as well as predictive of health outcomes [4, 9]. The sex-specific data from this study cohort with DXA measures concurs with known literature showing mean and median values for total LM in men are higher than seen in women across all age groups [7, 12, 19]. The current results showed a reduction in mean LM from the 20–29 year age group through the 70–79 year age group of approximately 2% and 3% per decade in women and men, respectively. The greater reduction per decade in men causes the difference in total LM between sexes to narrow with age, from a mean sex difference of 21.9 kg (percent difference: 33.5%) in the youngest age group to a mean sex difference of 15.5 kg (percent difference: 27.9%) in the oldest age group. Compared to the Mexican and Australian cohorts the women in the current study had higher mean total LM at each age group [7, 19]. The mean total LM in men from the current study was higher at each age group compared to men in the Mexican cohort and was higher up until the 70–79 year age group compared to men of the Australian cohort. The reduction in total LM across each decade was also different between the cohorts. Men in this cohort had a greater reduction per decade than both the Mexican and Australian men [7, 19]. The reductions in total LM per decade in women were similar between our cohort and the Australian women (both cohorts showed reductions of approximately 2% per decade) but was higher than that reported in Mexican women (0.5% reduction per decade) [7, 19].

DXA is capable of separating soft tissue into fat and lean mass components, thereby permitting the evaluation of LM without the confounding influence of other tissue constituents, such as excess fat. As a result, LMI has been a useful measure in identifying those with LM deficits and at risk of sarcopenia and/or physical disability [27]. Data from our study sample reveal that median LMI is significantly reduced from the youngest (20–29 years) to oldest (70–79 years) age groups in both men and women. Although no formal statistical analysis was performed, it was observed that the median values from the current study for LMI were lower across all age groups in women and in all age groups after the youngest age group (20–29 years) in men compared to the NHANES dataset [12]. Although these differences could have stemmed from differences in the recruitment processes, both study samples provided a large distribution of US adults across a wide age-range, however the NHANES cohort was recruited from a larger geographical compared to the current study. That being said, these comparisons are consistent with the results reported by Shepherd et al. who found that whole-body LM measurement results from GE-Healthcare DXA systems were lower than Hologic systems [15]. However, our LMI reference values were higher than those proposed by Fan et al. using the cross-calibrated prediction equation between Hologic and GE-Healthcare models [18]. The observed differences between sex and age-group reference values using the GE-Healthcare DXA systems compared to those created using Hologic or prediction equations are not meant to downplay the use of these references values when appropriate, but instead emphasize the need for instrumentation specific and directly measured reference values.

Using the LMI classification ranges developed by Janssen et al. to help diagnose sarcopenia, there were no men or women from the current cohort that were identified as having sarcopenia or at risk for physical disability (< 10.75 kg m^-2^ and 5.75 kg m^-2^, respectively) [9], which may be a result of the difference in the body composition method used in deriving these cut-points, and/or the current study sample being apparently healthy men and women.

Median %LM was significantly higher in men across all age groups. In both men and women, median %LM decreased with age up until the 50–59 year age group, after which there was an increasing trend in %LM. This increasing trend in the older age groups (60–69 and 70–79), may again be due to the apparently healthy study sample, rather than a clinical population.

Reference values for ALMI were developed as this measure is a good indicator of skeletal muscle mass and is commonly used in defining sarcopenia [8, 10, 27]. In both men and women, there was a decreasing trend in mean ALMI with age with a mean absolute difference between the youngest and oldest age groups of 1.53 and 0.63 kg m^-2^, respectively. Similar to median LMI values, the median values for ALMI were higher than those proposed by Fan et al. using the cross-calibrated equations in both men and women. Additionally, the median values for ALMI were lower in the men and women across all age groups in this study sample, compared to the NHANES cohort [12]. Again, this may be attributed to the differences in the specific instrumentation used.

Diagnostic criteria to define sarcopenia have used cutoff points for ALMI of > 2SD below the young reference population (18–39 years of age) [8, 12, 27], as well as below the 20^th^ percentile in an older adult population of similar age (≥70 years) [10]. Cutoff points for ALMI using the established diagnostic criteria of >2SD below the young reference population, were 6.35 kg m^-2^ for men and 4.92 kg m^-2^ for women in this study cohort. These cutoff points are lower than those derived from the Australian adult cohort (6.94 kg m^-2^ men, 5.30 kg m^-2^ women) and cutoff points from a different US non-Hispanic white population (7.26 kg m^-2^ men, 5.45 kg m^-2^ women), both of which were measured using GE-Healthcare DXA systems [7, 8]. The current study’s cutoff points may differ from the Australian cohort due to geographical differences between the two study samples [7]. The higher cutoff points reported in the other US non-Hispanic white population may be due to their smaller sample size and/or representativeness of their young adult sample which the authors declare as “unknown” [8]. When using the criteria of ALMI >2SD below the young reference standard to define sarcopenia (6.35 kg m^-2^ in men, 4.92 kg m^-2^ in women), 17 men and 24 women would be classified as sarcopenic, of which only included 3 men and 2 women from the 70–79 year age group. However when using the criteria of ≤ 20^th^ percentile for ALMI in an older adult population (defined as over age 70 years) higher cutoff points for defining sarcopenia were found in the current study (7.40 kg m^-2^ in men, 5.60 kg m^-2^ in women). These cutoff points using the ≤ 20^th^ percentile criteria are similar to those derived from Delmonico et al. who studied 2,976 US adults and reported cutoff points of 7.25 and 5.67 kg m^-2^ in men and women for defining sarcopenia [10]. When the current study’s cutoff points using ≤20^th^ percentile were applied, 34 men and 32 women in the 70–79 year age group were classified as sarcopenic. The discrepancy in the amount of individuals classified as sarcopenic by criteria used highlights the importance of developing standardized diagnostic criteria for defining sarcopenia (Table 6 provides a comparison of different measurable LM variables and their cut-points used in defining sarcopenia).

**Table 6:** Measurable lean mass variables and cut-off points used for the diagnosis of sarcopenia.

**Criterion Method**	**Reference**	**Measurement Method**	**Established cut-points by gender**	*New cut-points by gender	**Participants meeting established cut- points (n, %)**	*Participants meeting new cut-points (n, %)
**Lean mass (kg)**	Castillo et al [11]	Bioelectrical Impedance: (Valhalla Med., Model 1990B) ≥ 2 SD below mean of young reference population (18-39y)	**Men:** ≤ 47.9 kg **Women:** ≤ 34.7 kg	**Men:** ≤43.5 **Women:** ≤30.5	**Men:** (55, 4.4%) **Women:** (178, 8.6%)	**Men:** (16, 1.3%) **Women**: (12, 0.6%)
**Lean Mass Index**	Janssen et al [9, 28]	Bioelectrical Impedance: Valhalla Med., Model 1990B ROC analysis was used to develop cut-points associated with moderate and high physical disability	**Men** Moderate: 10.75 kg m^-2^ High: 8.50 kg m^-2^ **Women** Moderate: 6.75 kg m^-2^ High: 5.75 kg m^-2^	+	**Men:** (0, 0%) **Women:** (0, 0%)	+
**Percent Lean Mass**	Janssen et al [28]	Bioelectrical Impedance: (Valhalla Med., Model 1990B) Class I: 1–2 SD below reference population (18-39y) Class II: > 2 SD below reference population	**Men** Class I: 37% Class II: 31% **Women** Class I: 28% Class II: 22%	**Men** Class I: 56.4% Class II:47.4% **Women** Class I:46.0% Class II: 36.8%	**Men** Class I: (0, 0%) Class II: (0, 0%) **Women** Class I: (0, 0%) Class II: (0, 0%)	**Men** Class I: (69, 5.5%) Class II: (11, 0.9%) **Women** Class I: (136, 6.6%) Class II: (1, 0.1%)
**Appendicular Lean Mass Index**	Baumgartner et al Melton et al [8, 29] Delmonico et al [10]	DXA: (Lunar DPX [8], Hologic QDR 2000[29], Hologic QDR 4500 A [10]) ≥ 2 SD below mean young reference population (18-39y) Or > 20^th^ % ile of specific gender and aged population	**Men:** 7.26 kg m^-2^ **Women:** 5.50 kg m^-2^ **Men:** 7.25 kg m^-2^ **Women:** 5.67 kg m^-2^ (70–79 years of age)	**Men**: 6.35 kg m^-2^ **Women:** 4.92 kg m^-2^ **Men:** 7.40 kg m^-2^ **Women:** 5.60 kg m^-2^ (70–79 year age group)	**Men:** (95, 7.6%) **Women:** (162, 7.8%) **Men:** (27, 2.2%) **Women:** (35, 1.6%)	**Men:** (17, 1.4%) **Women:** (24, 1.2%) **Men ≥ 70 y**: (34, 2.7%) **Women ≥ 70 y:** (32, 1.5%)

*New cut-points were generated using the same measurement criteria as the established cut-points, but using the current study population.

+ New cut-points were unable to be generated for lean mass index using the current study population.

This study is not without limitations. First, the current study cohort was 95% white. Ethnic variation in body composition has been documented in the literature [30, 31], and therefore these reference ranges may not be an accurate representation for all ethnic groups. All participants in this cohort were apparently healthy and therefore reference values for specific clinical populations are needed. Additionally, the GE lunar prodigy and iDXA have weight limits of 159 (350 lb.) and 204 kg (450 lb.), respectively and therefore these data may not be representative of morbidly obese that weigh ≥159 kg (350 lb.) or ≥204 kg (450lb.). Additionally, we could not statistically compare our population sample to the NHANES cohort and therefore we cannot rule out that differences between the current reference values using the GE-Healthcare DXA systems compared to those created using Hologic or prediction equations were not in part due to population differences. This study had several strengths including using pooled data from two laboratories with standardized procedures and a subject group with a wide-range of characteristics including age, BMI, and physical activity levels.

### Conclusion

Body composition measurements are recognized as an important tool in research and clinical settings to assess health and nutritional status. DXA scans provide a high-quality measure of both whole-body and regional body composition measures, however, the interpretation of DXA data has been limited by lack of reference values. The results from this study provide directly measured reference values for total LM, LMI, %LM, and ALMI using the GE-Healthcare models. These reference values will provide GE-Healthcare DXA system users with the ability to derive meaningful interpretation of the results obtained from whole-body and regional DXA scans. As a result, the proposed reference values will also help in the early detection of those at an increased risk of sarcopenia, frailty, and/or physical disability, and lead to early and appropriate interventions to minimize LM deficits.

## Supporting information

S1 Fig Lean mass (kg) vs. age in women. Lines indicate 3^rd^ (black), 50^th^ (red), and 97^th^ (green) percentiles.
(TIF)

S2 Fig Lean mass (kg) vs. age in men. Lines indicate 3^rd^ (black), 50^th^ (red), and 97^th^ (green) percentiles.
(TIF)

S3 Fig Lean mass index (kg m^-2^) vs. age in women. Lines indicate 3^rd^ (black), 50^th^ (red), and 97^th^ (green) percentiles.
(TIF)

S4 Fig Lean mass index (kg m^-2^) vs. age in men. Lines indicate 3^rd^ (black), 50^th^ (red), and 97^th^ (green) percentiles.
(TIF)

S5 Fig Percent lean mass (%) vs. age in women. Lines indicate 3^rd^ (black), 50^th^ (red), and 97^th^ (green) percentiles.
(TIF)

S6 Fig Percent lean mass (%) vs. age in men. Lines indicate 3^rd^ (black), 50^th^ (red), and 97^th^ (green) percentiles.
(TIF)

S7 Fig Appendicular lean mass index (kg m^-2^) vs. age in women. Lines indicate 3^rd^ (black), 50^th^ (red), and 97^th^ (green) percentiles.
(TIF)

S8 Fig Appendicular lean mass index (kg m^-2^) vs. age in men. Lines indicate 3^rd^ (black), 50^th^ (red), and 97^th^ (green) percentiles.
(TIF)

S1 Table Lean mass vs. age-group in women. 3rd, 50th, and 97th percentile values for total lean mass in women for smoothed age-group values.
(PDF)

S2 Table Lean mass vs. age-group in men. 3rd, 50th, and 97th percentile values for total lean mass in men for smoothed age-group values.
(PDF)

S3 Table Lean mass index vs. age-group in women. 3rd, 50th, and 97th percentile values for lean mass index in women for smoothed age-group values.
(PDF)

S4 Table Lean mass index vs. age-group in men. 3rd, 50th, and 97th percentile values for lean mass index in men for smoothed age-group values.
(PDF)

S5 Table Percent lean mass vs. age-group in women. 3rd, 50th, and 97th percentile values for percent lean mass in women for smoothed age-group values.
(PDF)

S6 Table Percent lean mass vs. age-group in men. 3rd, 50th, and 97th percentile values for percent lean mass in men for smoothed age-group values.
(PDF)

S7 Table Appendicular lean mass index vs. age-group in women. 3rd, 50th, and 97th percentile values for appendicular lean mass index in women for smoothed age-group values.
(PDF)

S8 Table Appendicular lean mass index vs. age-group in men. 3rd, 50th, and 97th percentile values for appendicular lean mass index in men for smoothed age-group values.
(PDF)
