# Supplementary figures and images for: Reference standards for lean mass measures using GE dual energy x-ray absorptiometry in Caucasian adults
Source: PLoS One. 2017 Apr 20;12(4):e0176161. doi: 10.1371/journal.pone.0176161 (PMC5398591; doi:10.1371/journal.pone.0176161)

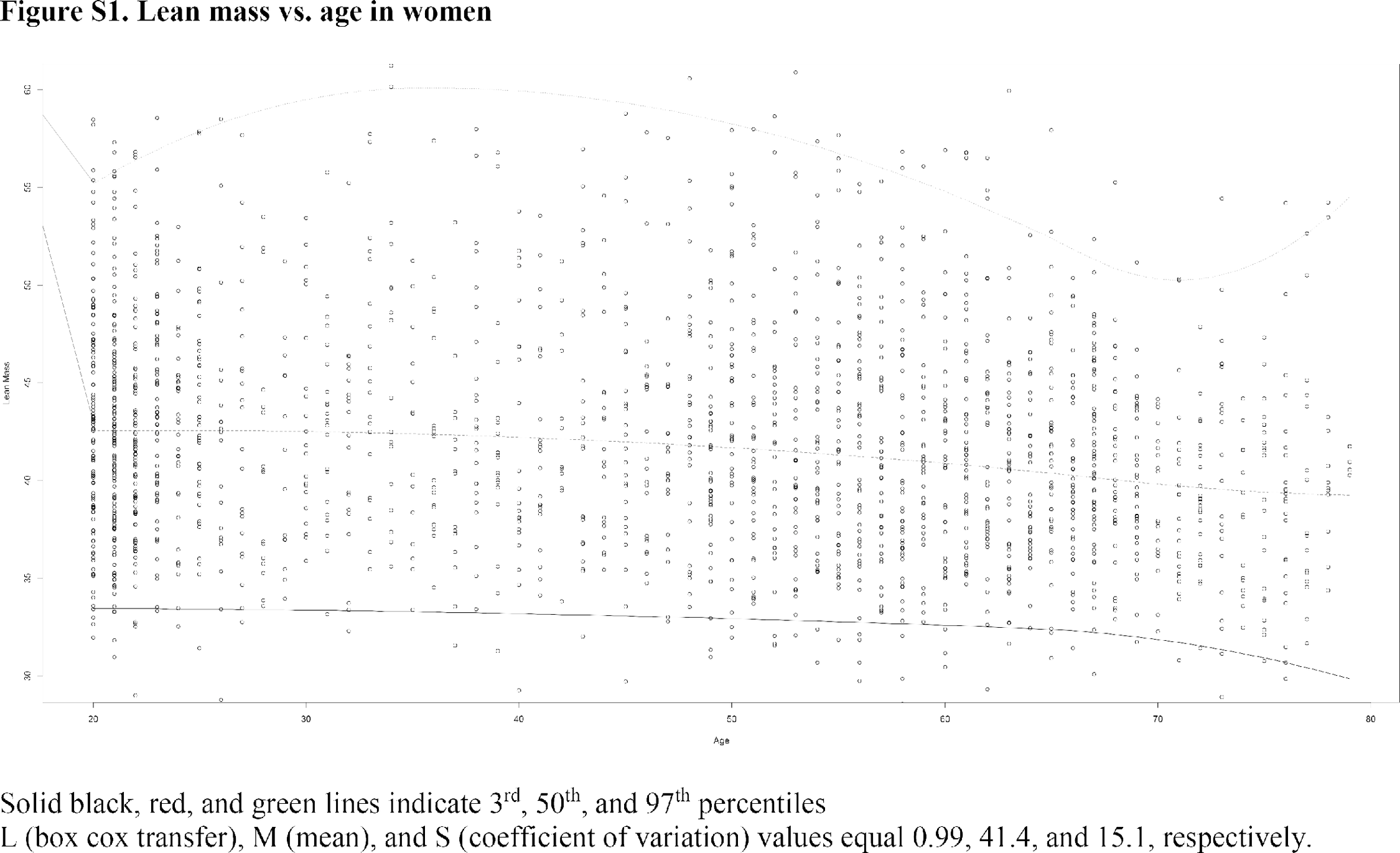

Supplement: S1 Fig — Lines indicate 3rd (black), 50th (red), and 97th (green) percentiles. (TIF) [file pone.0176161.s001.tif]

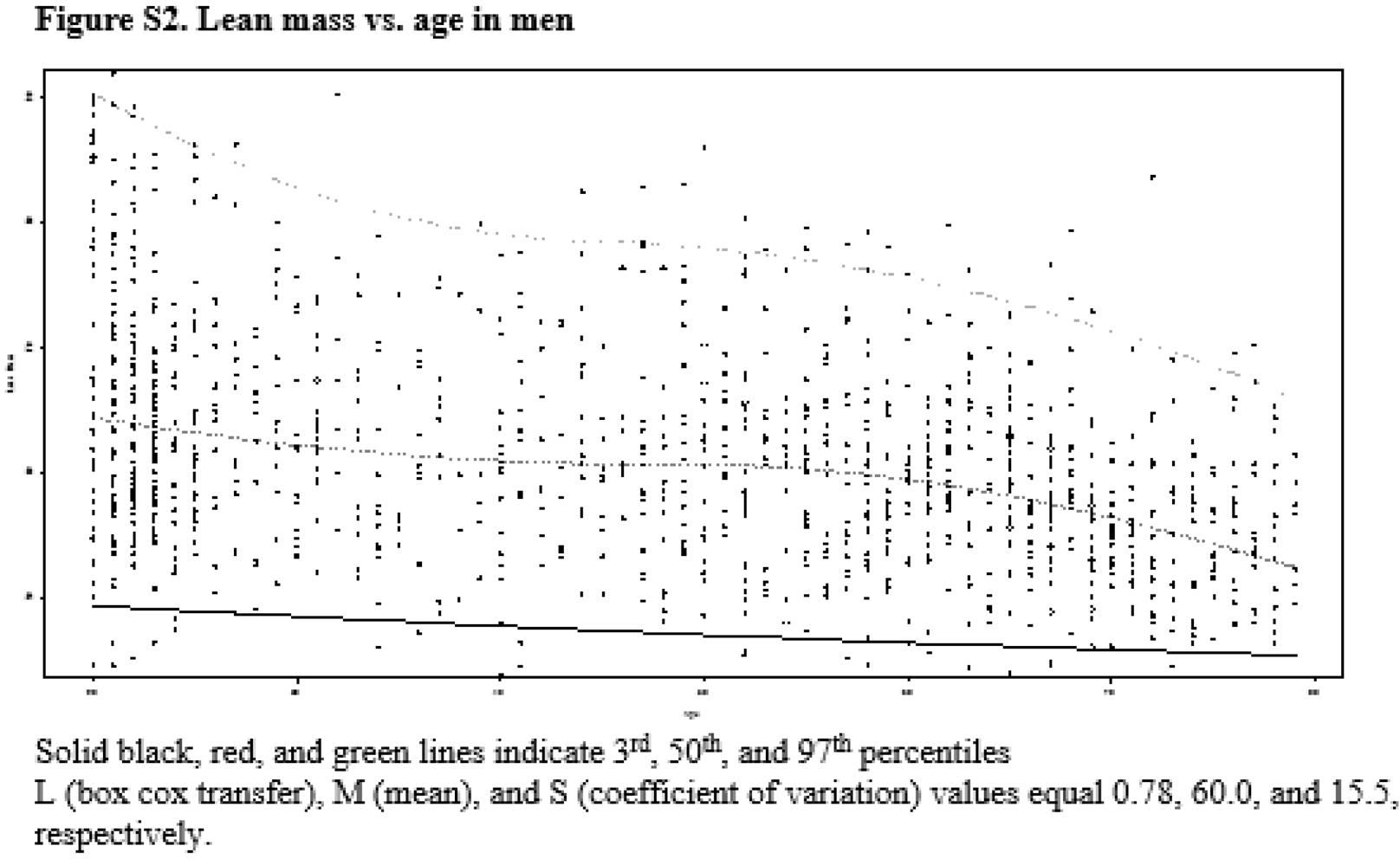

Supplement: S2 Fig — Lines indicate 3rd (black), 50th (red), and 97th (green) percentiles. (TIF) [file pone.0176161.s002.tif]

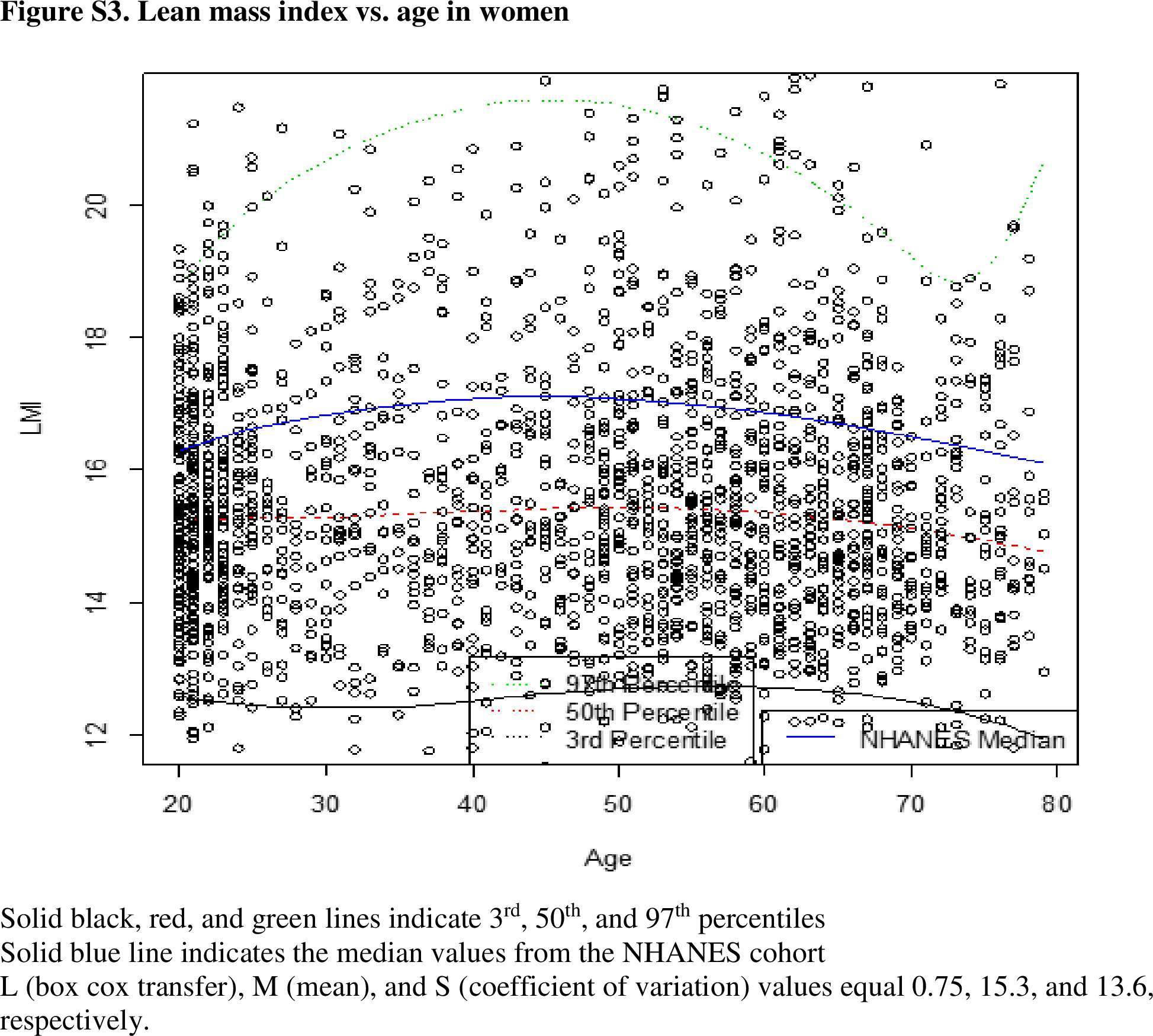

Supplement: S3 Fig — Lines indicate 3rd (black), 50th (red), and 97th (green) percentiles. (TIF) [file pone.0176161.s003.tif]

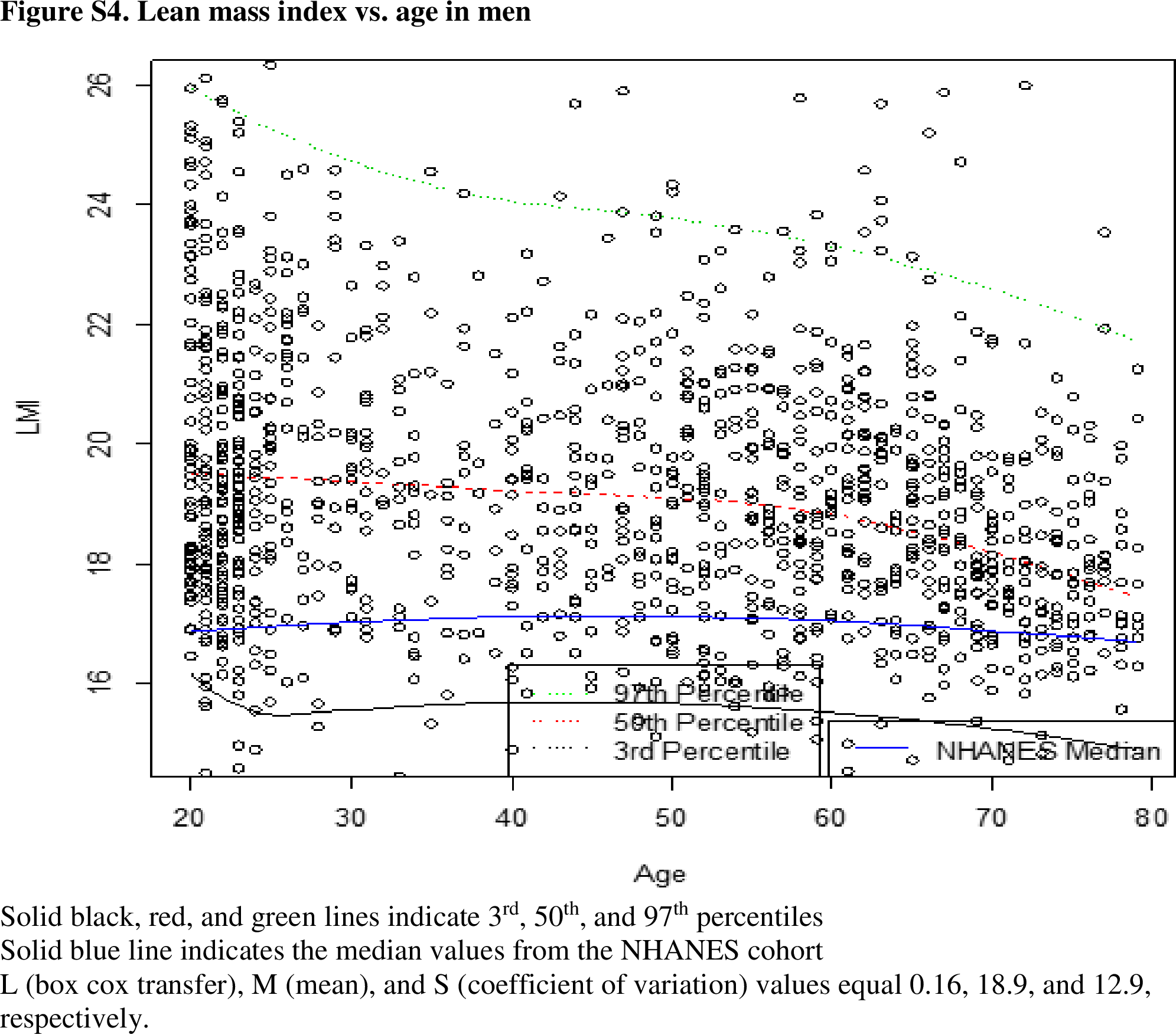

Supplement: S4 Fig — Lines indicate 3rd (black), 50th (red), and 97th (green) percentiles. (TIF) [file pone.0176161.s004.tif]

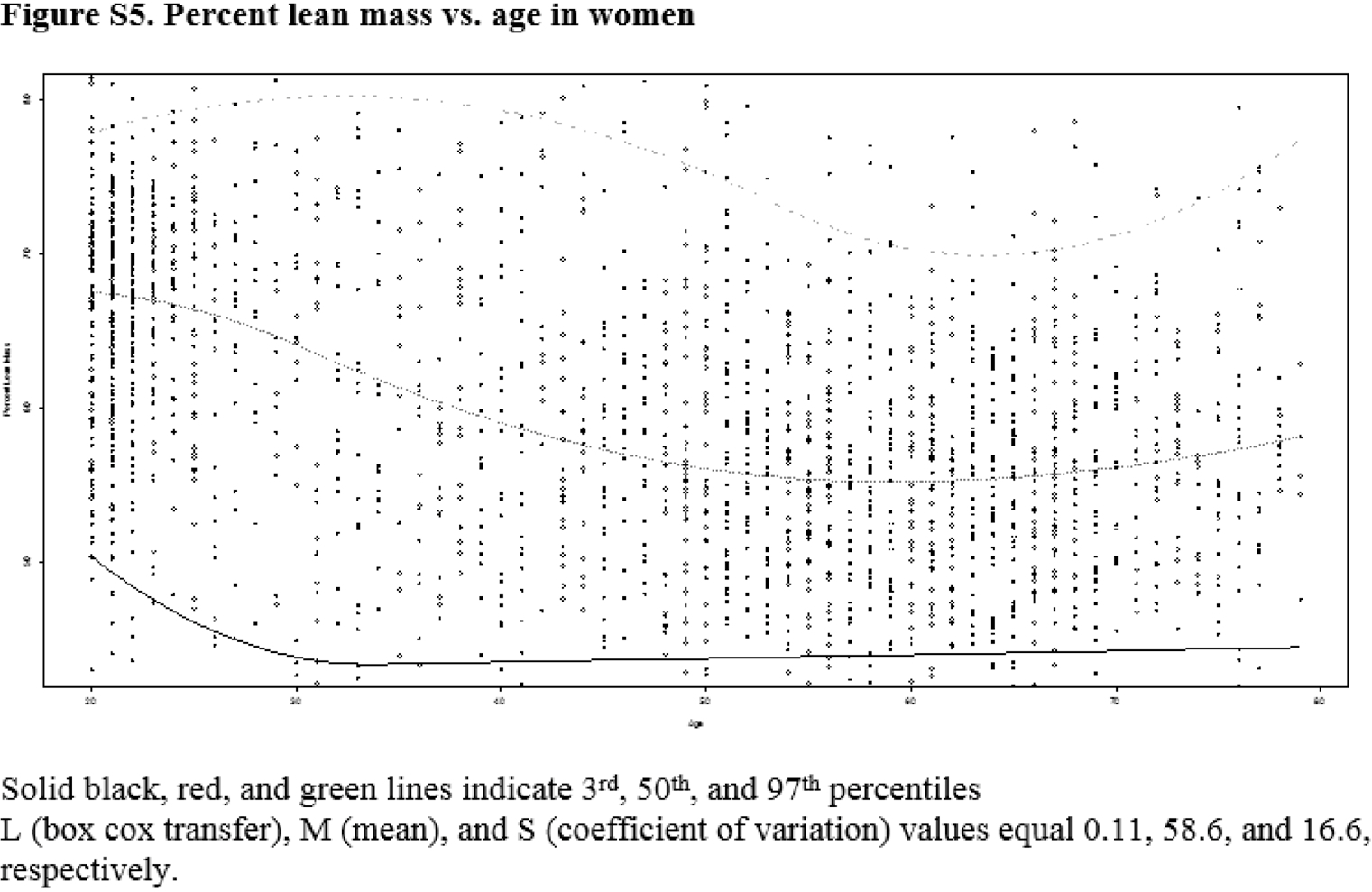

Supplement: S5 Fig — Lines indicate 3rd (black), 50th (red), and 97th (green) percentiles. (TIF) [file pone.0176161.s005.tif]

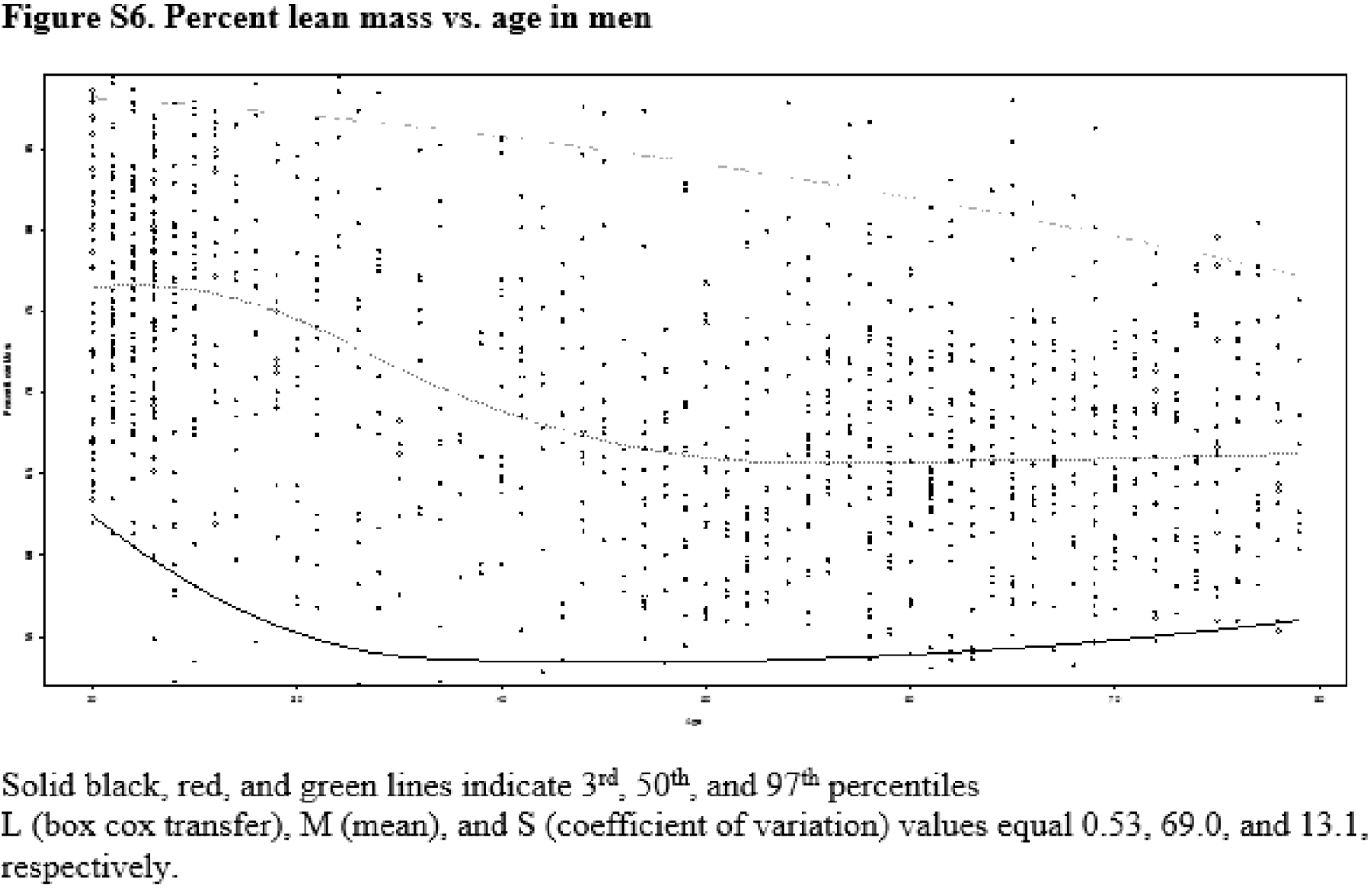

Supplement: S6 Fig — Lines indicate 3rd (black), 50th (red), and 97th (green) percentiles. (TIF) [file pone.0176161.s006.tif]

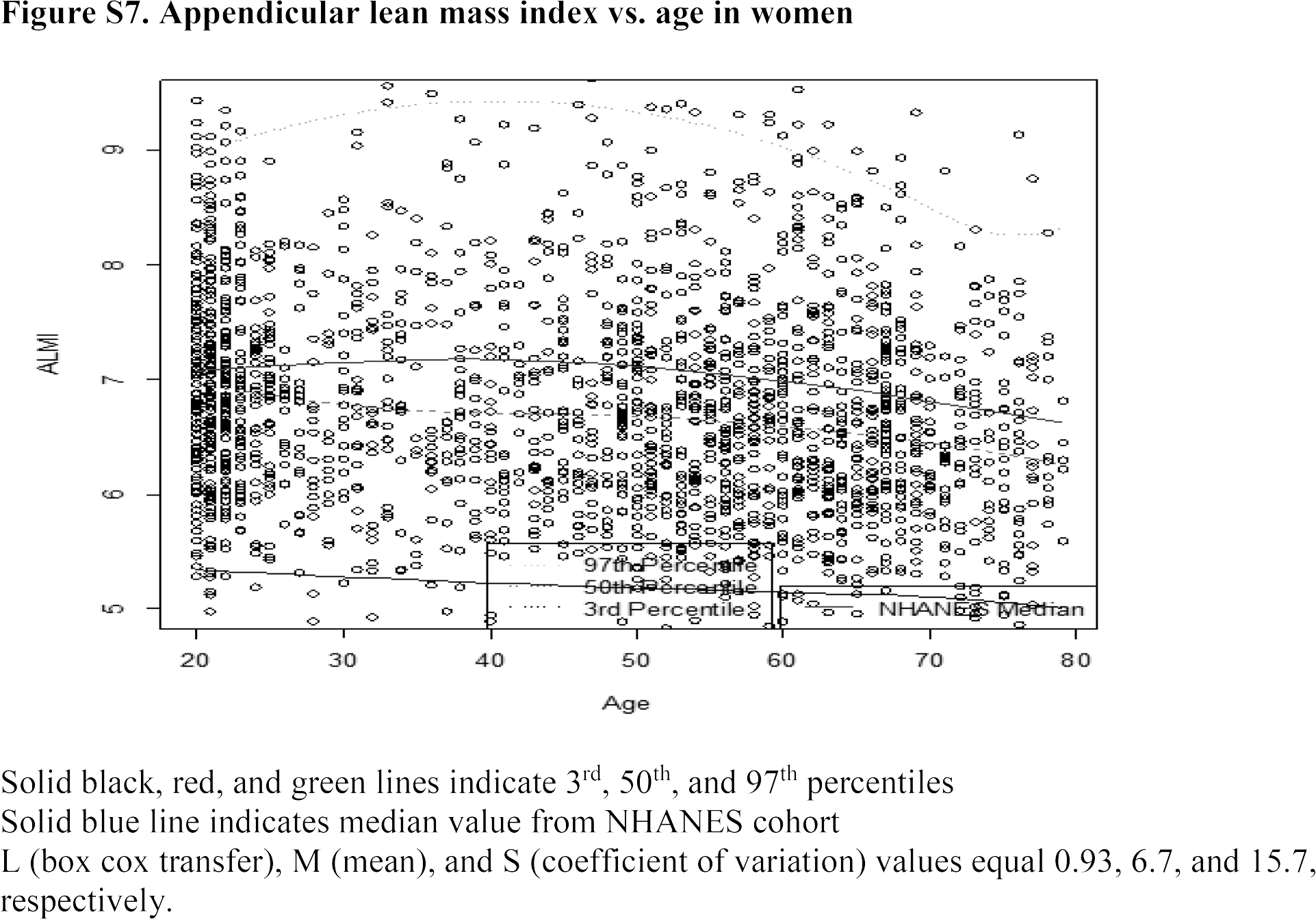

Supplement: S7 Fig — Lines indicate 3rd (black), 50th (red), and 97th (green) percentiles. (TIF) [file pone.0176161.s007.tif]

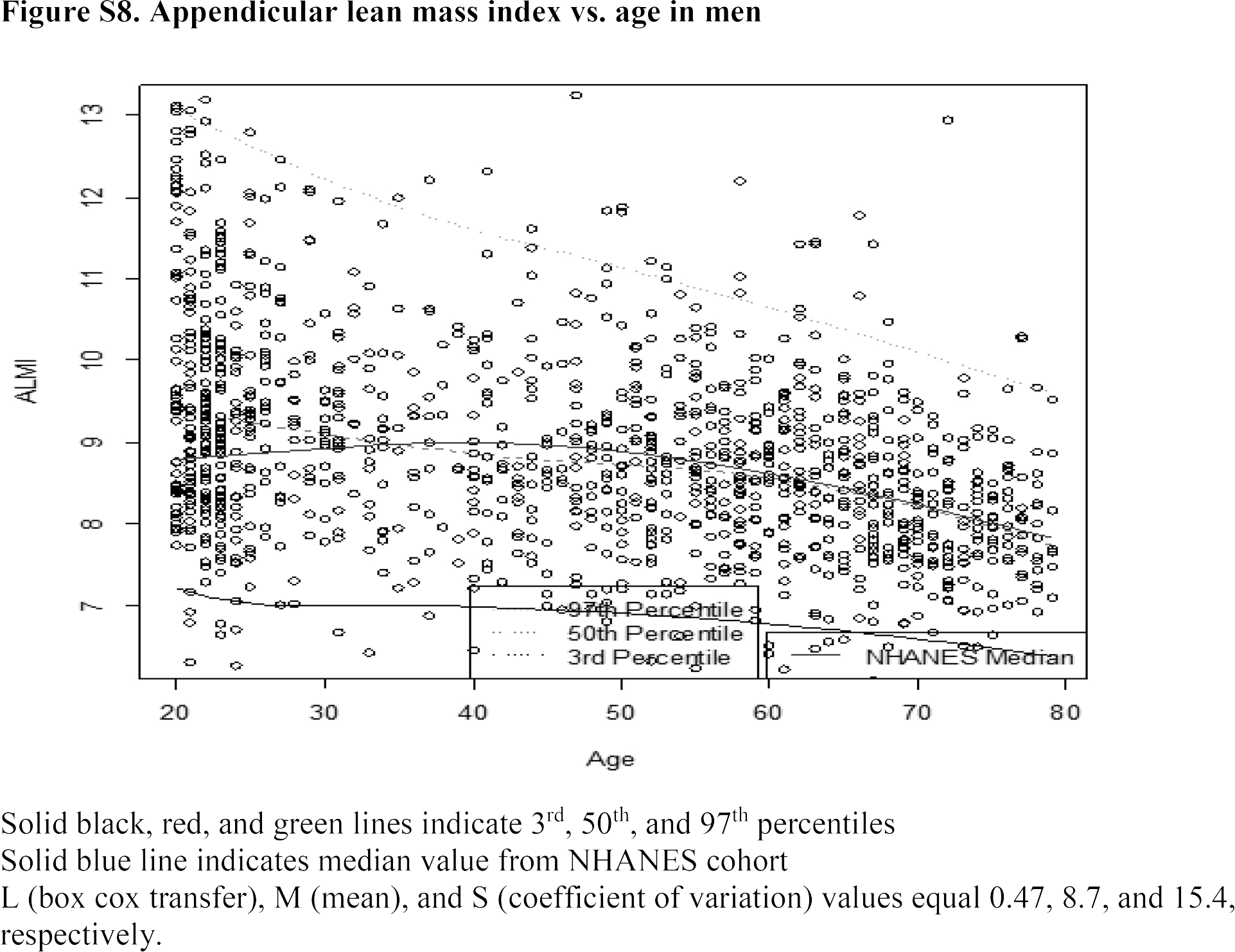

Supplement: S8 Fig — Lines indicate 3rd (black), 50th (red), and 97th (green) percentiles. (TIF) [file pone.0176161.s008.tif]
