# Supplementary material for: Reference standards for lean mass measures using GE dual energy x-ray absorptiometry in Caucasian adults
Source: PLoS One. 2017 Apr 20;12(4):e0176161. doi: 10.1371/journal.pone.0176161 (PMC5398591; doi:10.1371/journal.pone.0176161)
Supplement: S1 Table — 3rd, 50th, and 97th percentile values for total lean mass in women for smoothed age-group values. (PDF) [file pone.0176161.s009.pdf]

Table S1. Lean mass vs. age-group in women

Smoothed age-group	3%	50%	97%
1	33.44789	42.51281	53.79747
2	33.44249	42.53044	54.83637
3	33.43521	42.54246	55.78188
4	33.42605	42.54888	56.63399
5	33.41501	42.54970	57.39270
6	33.40208	42.54491	58.05801
7	33.38727	42.53451	58.62993
8	33.37058	42.51852	59.10844
9	33.35201	42.49691	59.49356
10	33.33155	42.46970	59.78527
11	33.30922	42.43689	59.98359
12	33.28499	42.39848	60.08851
13	33.25889	42.35445	60.10003
14	33.23091	42.30483	60.07105
15	33.20104	42.24960	60.00156
16	33.16929	42.18876	59.89158
17	333.13565	42.12232	59.74109
18	33.10014	42.05028	59.55010
19	33.06274	41.97263	59.31860
20	33.02346	41.88938	59.04661
21	32.98230	41.80052	58.73411
22	32.93925	41.70551	58.38111
23	32.89432	41.60434	57.98761
24	32.84751	41.49701	57.55360
25	32.79882	41.38353	57.07910
26	32.74825	41.26389	56.56409
27	32.69579	41.13810	56.00858
28	32.64145	41.00615	55.41256
29	32.58523	40.86804	54.77605
30	32.52712	40.72378	54.09903
31	32.46713	40.57336	53.38151
32	32.40526	40.41678	52.62349
33	32.30498	40.25405	51.82496
34	32.16628	40.08517	50.98593
35	31.98917	39.91012	50.44792
36	31.77364	39.75408	50.21090
37	31.51970	39.61704	50.27490
38	31.22734	39.49899	50.63990
39	30.89657	39.39995	51.30591
40	30.52738	39.31991	52.27293
41	30.11978	39.25887	53.54096
42	29.67377	39.21683	55.10999
43	29.18934	39.19380	56.98003
