# Supplementary material for: Reference standards for lean mass measures using GE dual energy x-ray absorptiometry in Caucasian adults
Source: PLoS One. 2017 Apr 20;12(4):e0176161. doi: 10.1371/journal.pone.0176161 (PMC5398591; doi:10.1371/journal.pone.0176161)
Supplement: S2 Table — 3rd, 50th, and 97th percentile values for total lean mass in men for smoothed age-group values. (PDF) [file pone.0176161.s010.pdf]

Table S2. Lean mass vs. age-group in men

Smoothed age-group	3%	50%	97%
1	49.49685	64.93516	92.32127
2	49.36389	64.51158	90.84131
3	49.23249	64.10967	89.44482
4	49.10263	63.72943	88.13182
5	48.97432	63.37085	86.90230
6	48.84756	63.03394	85.75627
7	48.72235	62.71870	84.69372
8	48.59869	62.42512	83.71466
9	48.47657	62.15322	82.81907
10	48.35600	61.90297	82.00698
11	48.23698	61.67440	81.27836
12	48.11951	61.46749	80.63323
13	48.00358	61.28224	80.07158
14	47.88920	61.11867	79.59342
15	47.77637	60.97676	79.19874
16	47.66509	60.85652	78.88755
17	47.55536	60.75794	78.65983
18	47.44717	60.68103	78.51561
19	47.34053	60.62579	78.45486
20	47.23544	60.59221	78.35208
21	47.13190	60.58030	78.20726
22	47.02991	60.59006	78.02040
23	46.92946	60.55519	77.79150
24	46.83056	60.47569	77.52056
25	46.73321	60.35155	77.20758
26	46.63741	60.18279	76.85256
27	46.54315	59.96940	76.45551
28	46.45044	59.71137	76.01642
29	46.35928	59.40872	75.53528
30	46.26967	59.06143	75.01211
31	46.18161	58.66952	74.44690
32	46.09509	58.23297	73.83965
33	46.01013	57.75180	73.19037
34	45.92671	57.22599	72.49904
35	45.84483	56.67478	71.76568
36	45.76451	56.09815	70.99027
37	45.68573	55.49612	70.17283
38	45.60850	54.86869	69.31335
39	45.53282	54.21584	68.41183
40	45.45869	53.53759	67.46827
41	45.38611	52.83393	66.48267
42	45.31507	52.10486	65.45504
43	45.24558	51.35039	64.38536
