# Supplementary material for: Reference standards for lean mass measures using GE dual energy x-ray absorptiometry in Caucasian adults
Source: PLoS One. 2017 Apr 20;12(4):e0176161. doi: 10.1371/journal.pone.0176161 (PMC5398591; doi:10.1371/journal.pone.0176161)
Supplement: S3 Table — 3rd, 50th, and 97th percentile values for lean mass index in women for smoothed age-group values. (PDF) [file pone.0176161.s011.pdf]

Table S3. Lean mass index vs. age-group in women

Smoothed age-group	3%	50%	97%
1	12.64257	15.23493	18.08964
2	12.59092	15.23951	18.59552
3	12.54552	15.24483	19.04544
4	12.50637	15.25089	19.43938
5	12.47347	15.25770	19.77736
6	12.44683	15.26526	20.05936
7	12.42644	15.27356	20.28540
8	12.41230	15.28261	20.49369
9	12.40442	15.29240	20.68425
10	12.40279	15.30294	20.85707
11	12.40741	15.31422	21.01214
12	12.41828	15.32625	21.14948
13	12.43541	15.33902	21.26908
14	12.45879	15.35254	21.37093
15	12.48842	15.36681	21.45505
16	12.52230	15.38182	21.52143
17	12.56043	15.39757	21.57006
18	12.59467	15.41407	21.60096
19	12.62501	15.42702	21.61412
20	12.65145	15.43641	21.60953
21	12.67401	15.44225	21.58721
22	12.69266	15.44453	21.54715
23	12.70743	15.44326	21.48934
24	12.71830	15.43844	21.41380
25	12.72527	15.43006	21.32052
26	12.72835	15.41813	21.20950
27	12.72754	15.40264	21.08073
28	12.72283	15.38360	20.93423
29	12.71423	15.36100	20.76999
30	12.70174	15.33486	20.58801
31	12.68535	15.30515	20.38828
32	12.66506	15.27189	20.17082
33	12.63450	15.23508	19.93562
34	12.59364	15.19472	19.68268
35	12.54250	15.15079	19.41200
36	12.48108	15.10332	19.12357
37	12.40937	15.05229	18.86226
38	12.32738	14.99771	18.83353
39	12.23510	14.93957	19.03738
40	12.13254	14.87788	19.47380
41	12.01969	14.81263	20.14282
42	11.89656	14.74383	21.04441
43	11.76314	14.67148	22.17858
