# Supplementary material for: Reference standards for lean mass measures using GE dual energy x-ray absorptiometry in Caucasian adults
Source: PLoS One. 2017 Apr 20;12(4):e0176161. doi: 10.1371/journal.pone.0176161 (PMC5398591; doi:10.1371/journal.pone.0176161)
Supplement: S4 Table — 3rd, 50th, and 97th percentile values for lean mass index in men for smoothed age-group values. (PDF) [file pone.0176161.s012.pdf]

Table S4. Lean mass index vs. age-group in men

Smoothed age-group	3%	50%	97%
1	16.75930	19.54442	26.28931
2	16.30253	19.52153	26.05134
3	15.94407	19.49884	25.82598
4	15.68391	19.47636	25.61322
5	15.52205	19.45409	25.41307
6	15.45850	19.43203	25.22552
7	15.49325	19.41017	25.05059
8	15.52507	19.38852	24.88825
9	15.55395	19.36707	24.73853
10	15.57990	19.34584	24.60141
11	15.60292	19.32480	24.47690
12	15.62300	19.30398	24.36499
13	15.64015	19.28336	24.26569
14	15.65437	19.26295	24.17900
15	15.66565	19.24275	24.10491
16	15.67400	19.22275	24.04343
17	15.67942	19.20296	23.99455
18	15.68190	19.18337	23.95829
19	15.68145	19.16400	23.92649
20	15.67806	19.14483	23.88917
21	15.67175	19.12586	23.84632
22	15.66249	19.10710	23.79793
23	15.65031	19.08855	23.74402
24	15.63519	19.06223	23.68458
25	15.61713	19.02812	23.61961
26	15.59615	18.98624	23.54911
27	15.57223	18.93658	23.47308
28	15.54537	18.87915	23.39152
29	15.51559	18.81393	23.30443
30	15.48287	18.74094	23.21182
31	15.44721	18.66018	23.11367
32	15.40862	18.57163	23.00999
33	15.36710	18.47531	22.90079
34	15.32265	18.37121	22.78605
35	15.27526	18.25934	22.66578
36	15.22494	18.14391	22.53999
37	15.17168	18.02492	22.40866
38	15.11549	17.90238	22.27181
39	15.05637	17.77628	22.12943
40	14.99431	17.64663	21.98151
41	14.92932	17.51342	21.82807
42	14.86140	17.37666	21.66910
43	14.79054	17.23634	21.50460
