# Supplementary material for: Reference standards for lean mass measures using GE dual energy x-ray absorptiometry in Caucasian adults
Source: PLoS One. 2017 Apr 20;12(4):e0176161. doi: 10.1371/journal.pone.0176161 (PMC5398591; doi:10.1371/journal.pone.0176161)
Supplement: S5 Table — 3rd, 50th, and 97th percentile values for percent lean mass in women for smoothed age-group values. (PDF) [file pone.0176161.s013.pdf]

Table S5. Percent lean mass vs. age-group in women

Smoothed age-group	3%	50%	97%
1	52.67357	67.67939	77.02844
2	50.96087	67.60379	77.66647
3	49.42266	67.42485	78.23376
4	48.05894	67.14257	78.73032
5	46.86971	66.75694	79.15615
6	45.85497	66.26797	79.51124
7	45.01471	65.67566	79.79559
8	44.34895	64.98001	80.00921
9	43.85768	64.18101	80.15210
10	43.54089	63.27867	80.22425
11	43.39860	62.42415	80.22566
12	43.43080	61.61747	80.15634
13	43.46328	60.85861	80.01629
14	43.49606	60.14757	79.80550
15	43.52913	59.48437	79.52398
16	43.56250	58.86898	79.17172
17	43.59615	58.30143	78.74872
18	43.63010	57.78170	78.25499
19	43.66434	57.30980	77.69053
20	43.69887	56.88573	77.05533
21	43.73369	56.50948	76.34939
22	43.76880	56.18106	75.57272
23	43.80421	55.90047	74.72532
24	43.83990	55.66770	73.80718
25	43.87589	55.48276	72.81831
26	43.91217	55.34565	71.97310
27	43.94875	55.25637	71.27156
28	43.98561	55.21491	70.71369
29	44.02277	55.22127	70.29948
30	44.06022	55.27547	70.02894
31	44.09796	55.36040	69.90207
32	44.13599	55.47607	69.91887
33	44.17431	55.62248	70.07933
34	44.21293	55.79962	70.38346
35	44.25183	56.00751	70.83125
36	44.29103	56.24613	71.42272
37	44.33052	56.51549	72.15785
38	44.37031	56.81559	73.03665
39	44.41038	57.14643	74.05911
40	44.45075	57.50800	75.22524
41	44.49141	57.90031	76.53504
42	44.53236	58.32336	77.98851
43	44.57360	58.77715	79.58564
