# Supplementary material for: Reference standards for lean mass measures using GE dual energy x-ray absorptiometry in Caucasian adults
Source: PLoS One. 2017 Apr 20;12(4):e0176161. doi: 10.1371/journal.pone.0176161 (PMC5398591; doi:10.1371/journal.pone.0176161)
Supplement: S6 Table — 3rd, 50th, and 97th percentile values for percent lean mass in men for smoothed age-group values. (PDF) [file pone.0176161.s014.pdf]

Table S6. Percent lean mass vs. age-group in men

Smoothed age-group	3%	50%	97%
1	64.66618	75.99902	88.34622
2	63.02864	76.35008	88.22183
3	61.52201	76.54969	88.08988
4	60.14629	76.59787	87.95039
5	58.90149	76.49460	87.80334
6	57.78759	76.23989	87.64875
7	56.80462	75.83373	87.48660
8	55.95255	75.27613	87.31691
9	55.23140	74.56710	87.13967
10	54.64116	73.70661	86.95488
11	54.18183	72.69469	86.76254
12	53.85342	71.75095	86.56265
13	53.65592	70.87538	86.35521
14	53.58933	70.06800	86.14022
15	53.53349	69.32880	85.91768
16	53.48838	68.65778	85.68760
17	53.45402	68.05494	85.44996
18	53.43040	67.52028	85.20477
19	53.41752	67.05380	84.95204
20	53.41539	66.65550	84.69175
21	53.42399	66.32538	84.42392
22	53.44334	66.06345	84.14854
23	53.47343	65.86969	83.86560
24	53.51426	65.74411	83.57512
25	53.56584	65.68672	83.27709
26	53.62815	65.69750	82.97151
27	53.70121	65.71116	82.65838
28	53.78501	65.72770	82.33770
29	53.87955	65.74711	82.00947
30	53.98483	65.76939	81.67370
31	54.10086	65.79455	81.33037
32	54.22762	65.82258	80.97949
33	54.36513	65.85349	80.62107
34	54.51338	65.88728	80.25509
35	54.67237	65.92393	79.88157
36	54.84211	65.96347	79.50049
37	55.02258	66.00587	79.11187
38	55.21380	66.05116	78.71570
39	55.41576	66.09931	78.31198
40	55.62846	66.15035	77.90071
41	55.85190	66.20425	77.48189
42	56.08609	66.26104	77.05552
43	56.33102	66.32069	76.62160
