# Supplementary material for: Reference standards for lean mass measures using GE dual energy x-ray absorptiometry in Caucasian adults
Source: PLoS One. 2017 Apr 20;12(4):e0176161. doi: 10.1371/journal.pone.0176161 (PMC5398591; doi:10.1371/journal.pone.0176161)
Supplement: S7 Table — 3rd, 50th, and 97th percentile values for appendicular lean mass index in women for smoothed age-group values. (PDF) [file pone.0176161.s015.pdf]

Table S7. Appendicular lean mass index vs. age-group in women

Smoothed age-group	3%	50%	97%
1	5.353001	7.022948	8.874343
2	5.343033	6.983554	8.945923
3	5.333243	6.946745	9.012604
4	5.323632	6.912521	9.074387
5	5.314198	6.880884	9.131271
6	5.304942	6.851832	9.183258
7	5.295864	6.825365	9.230347
8	5.286965	6.801485	9.272537
9	5.278243	6.780190	9.309829
10	5.269699	6.761481	9.342223
11	5.261333	6.745357	9.369720
12	5.253145	6.731819	9.392318
13	5.245135	6.720867	9.410017
14	5.237303	6.712501	9.422819
15	5.229649	6.706720	9.430723
16	5.222173	6.703525	9.433728
17	5.214875	6.702916	9.431836
18	5.207754	6.700951	9.425045
19	5.200812	6.697630	9.413356
20	5.194048	6.692953	9.396769
21	5.187462	6.686921	9.375284
22	5.181054	6.679533	9.348901
23	5.174823	6.670789	9.317620
24	5.168771	6.660689	9.281440
25	5.162897	6.649234	9.240363
26	5.157200	6.636423	9.194387
27	5.151682	6.622256	9.143513
28	5.146341	6.606733	9.087741
29	5.141179	6.589855	9.027071
30	5.136194	6.571621	8.961503
31	5.131388	6.552031	8.891037
32	5.126759	6.531085	8.815673
33	5.122308	6.508784	8.735410
34	5.115869	6.485127	8.650250
35	5.107441	6.460114	8.560191
36	5.097024	6.433745	8.465234
37	5.084619	6.406020	8.365379
38	5.070225	6.376940	8.297127
39	5.053843	6.346504	8.260477
40	5.035471	6.314712	8.255430
41	5.015112	6.281565	8.281985
42	4.992763	6.247062	8.340143
43	4.968426	6.211203	8.429903
