# Supplementary material for: Reference standards for lean mass measures using GE dual energy x-ray absorptiometry in Caucasian adults
Source: PLoS One. 2017 Apr 20;12(4):e0176161. doi: 10.1371/journal.pone.0176161 (PMC5398591; doi:10.1371/journal.pone.0176161)
Supplement: S8 Table — 3rd, 50th, and 97th percentile values for appendicular lean mass index in men for smoothed age-group values. (PDF) [file pone.0176161.s016.pdf]

Table S8. Appendicular lean mass index vs. age-group in men

Smoothed age-group	3%	50%	97%
1	7.345694	9.461646	13.310016
2	7.245560	9.412304	13.155860
3	7.162526	9.364101	13.007216
4	7.096594	9.317039	12.864082
5	7.047762	9.271116	12.726459
6	7.016031	9.226332	12.594347
7	7.001402	9.182688	12.467746
8	7.003873	9.140184	12.346655
9	7.005121	9.098819	12.231076
10	7.005147	9.058595	12.121007
11	7.003949	9.019509	12.016449
12	7.001529	8.981564	11.917402
13	6.997886	8.944757	11.823866
14	6.993020	8.910463	11.735840
15	6.986931	8.878681	11.653326
16	6.979619	8.849411	11.576322
17	6.971084	8.822652	11.504829
18	6.961327	8.798406	11.438847
19	6.950346	8.776671	11.371800
20	6.938143	8.757449	11.303689
21	6.924717	8.740738	11.234513
22	6.910068	8.726539	11.164272
23	6.894196	8.714852	11.092967
24	6.877101	8.698997	11.020596
25	6.858783	8.678975	10.947161
26	6.839242	8.654784	10.872662
27	6.818479	8.626424	10.797097
28	6.796493	8.593897	10.720468
29	6.773283	8.557202	10.642774
30	6.748851	8.516339	10.564016
31	6.723196	8.471308	10.484193
32	6.696318	8.422109	10.403305
33	6.668218	8.368741	10.321352
34	6.638894	8.311206	10.238335
35	6.608348	8.249503	10.154253
36	6.576578	8.183631	10.069106
37	6.543586	8.113592	9.985124
38	6.509371	8.039385	9.902307
39	6.473933	7.961009	9.820656
40	6.437272	7.878466	9.740169
41	6.399388	7.791754	9.660847
42	6.360281	7.700874	9.582690
43	6.319952	7.605827	9.505698
